# Academic Stress and Burnout Reduction Through Mandala-Coloring and Grit-Enhancing: School-Based Interventions for Adolescents

**DOI:** 10.3390/bs15040439

**Published:** 2025-03-31

**Authors:** Xuening Fan, Anna Na Na Hui

**Affiliations:** Department of Social and Behavioral Sciences, City University of Hong Kong, Hong Kong SAR, China; xueninfan2-c@my.cityu.edu.hk

**Keywords:** academic stress, academic burnout, mandala, grit, school-based intervention

## Abstract

This quasi-experimental study evaluated the effectiveness of two school-based interventions aiming to reduce academic stress and alleviate burnout symptoms. Using cluster sampling, a total of 128 middle schoolers (*M*_age_ = 13.48, 42.2% female) from two classes in rural Henan Province, China, participated in this study. One class served as the control group (*n* = 61), while the other class was randomly assigned to two intervention groups: the mandala-coloring group (*n* = 31) and the grit-enhancing group (*n* = 32). ANCOVA and ANOVAs were conducted to detect any significant changes. The results showed that academic stress was significantly reduced in the mandala-coloring group (*F* = 5.741, *p* = 0.004, and *η^2^p* = 0.085), while no significant changes were observed in academic burnout. In the grit-enhancing group, a significant within-group increase in grit levels was found. These findings suggest that mandala coloring may serve as a time-efficient method for alleviating academic stress among adolescents. Implications for addressing academic stress and burnout in school settings are discussed.

## 1. Introduction

### 1.1. Academic Stress and Burnout

Academic stress has long been recognized worldwide as a hazardous factor leading to a series of negative outcomes, including poor performance, physical illness, sleeping problems, depression, and anxiety (e.g., Shankar & Park, 2016; Zhu et al., 2021). Comparing with their peers in Western countries, school-aged adolescents in Asian countries were reported to experience more academic stress mostly due to the influence of Confucian Heritage Culture, with the emphasis on hard work, the value of education, and filial piety to live up to family expectations (Tan & Yates, 2011). This is particularly evident in the context of China, as Zhao et al. (2015) pointed out that the test-oriented educational system in China further accented the overwhelming level of stress it imposed on students. The ranking practices exist at every level, from provinces and cities to schools and teachers, resulted in intense competitions, and the accompanied pressure at all levels is eventually shouldered by students. As they have further illustrated with examples, in addition to the countless assignments and tests faced in schools, students have to attend after-school tutoring with almost no time of their own (Zhao et al., 2015).

As aforementioned, this long-term excessive stress could largely affect students’ well-being, and one of the consequences is academic burnout (Gao, 2023). The concept of academic burnout was adapted from job burnout in the workplace, which was initially found as particularly prevalent among human services professionals (Maslach & Jackson, 1981). In parallel with job burnout, academic burnout is characterized by three sub-dimensions: emotional exhaustion (the state of being emotionally and physically worn by academic burdens), cynicism (the indifferent and doubtful attitude towards schoolwork), and inefficacy (the feeling of being incapable of completing schoolwork) (Schaufeli et al., 2002). As a reaction to prolongedly overbearing stress, academic burnout has been found to be positively associated with depressive symptoms (Cheng et al., 2020; Fiorilli et al., 2017). Researchers have explored possible interventions that could be applied within school settings to mitigate academic stress and burnout. Group counseling, mindfulness-based programs, time-management training, and exercise interventions were among some of the successful examples that have been carried out on Chinese students across different age groups (L. Tang et al., 2021).

### 1.2. Mindfulness-Based Interventions Tackle Academic Stress in School Settings

Mindfulness refers to the state of being deliberately attentive to one’s thoughts, feelings, and emotions at the present moment without judgment or evaluation (Kabat-Zinn, 2003). Such a particular state of moment-by-moment awareness provides relaxation to one’s body and mind, subsequently generating a positive impact on physical and psychological health (Amutio et al., 2015; Prazak et al., 2012). Mindfulness-based interventions (MBIs) have gained increasing research popularity in the past decade, with tons of studies showing MBIs as an effective method for relieving stress, anxiety, and depression across workplace and school settings (Zhang et al., 2021). Among various school-based interventions, MBIs have been recognized as one of the most effective approaches (van Loon et al., 2022).

The practices of MBIs come in forms of both formal and informal sessions. Two extensively recognized formal programs for mindfulness training include mindfulness-based cognitive therapy (MBCT) (Segal et al., 2018) and mindfulness-based stress reduction (MBSR) (Kabat-Zinn & Hanh, 2009). Generally, both programs are composed of approximately 2 h of weekly treatment over a duration of eight weeks, during which instructed meditative techniques along with assigned daily practice homework are delivered. The implementation of the two programs on student participants has displayed satisfying results in reducing stress and anxiety and promoting emotional stability (X. Liu, 2019; Song & Lindquist, 2015). Admittedly, though such promising outcomes are appealing, the time-consuming procedures may not be suitable for the already hectic academic life of Chinese middle schoolers. In addition to time constraints, several structural and implementation challenges have been identified. A meta-analysis by Emerson et al. (2020) highlighted multiple barriers, including fidelity issues due to inconsistencies in session structure and delivery, making it difficult to standardize and assess effectiveness. Additionally, the lack of trained facilitators poses a challenge, as an effective implementation relies on educators with formal mindfulness training, which many schools lack.

Taken together, these challenges underscore the need for more flexible, developmentally appropriate, and context-sensitive adaptations of MBIs in school settings. In this case, the advantages of informal, well-targeted sessions with short durations are explicit, and research showed that the informal mindfulness practices were as effective for maintaining positive mental well-being as the formal practices (Birtwell et al., 2019). The informal practices for mindfulness include, but are not limited to, performing daily mindful yoga and meditation, using mindfulness-based mobile apps, and engaging in mindfulness-oriented art activities (Fong et al., 2022; Gál et al., 2021; Schuver & Lewis, 2016).

In particular, art-based mandala coloring, as an easily accessible mindfulness tool, has been commonly used as a form of art therapy to develop a calm and meditative mood (Curry & Kasser, 2005). Unlike yoga or guided meditation, mandala coloring requires no special equipment, space, or trained instructors, making it ideal for schools with limited resources (Carsley et al., 2018). Beyond its logistical ease, mandala coloring offers psychological benefits by providing a structured yet creative activity. Research suggests that structured art activities, like mandala coloring, are more effective in reducing stress than free drawing, as they offer a balance between guidance and creative expression (Van Der Vennet & Serice, 2012).

A mandala refers to a circular shape with a symmetrical design. Studies have found that the movement of coloring a structured and intricate pattern is beneficial for restoring full concentration presently and distracting oneself from any other thoughts, and such meditative states could help relieve symptoms of negative emotions across occupations. For instance, in a 5-day mandala-based intervention, Czerwinski et al. (2021) suggested that the well-being of teachers from the UK in an intervention group had been significantly improved with lower levels of anxiety, stress, and burnout. Similarly, the effect of coloring mandalas was found to be significant in reducing perceived stress and boosting mental well-being throughout a 10-day intervention among Hong Kong nurses (Fong et al., 2022).

Specifically aiming at adolescents and young adults within school settings, mindfulness-based mandala coloring has also been applied effectively for alleviating test anxiety and academic stress (Apriyana et al., 2020; Carsley & Heath, 2018). Although empirical evidence supporting the effect of mandala coloring on academic burnout is lacking, the current study aimed to explore its potential impact, considering that burnout is the result of a build-up chronic stress.

### 1.3. Building Grit as a Protective Factor

In addition to the interventions that combat academic stress and burnout directly, some research took an alternative route by focusing on building resilience factors to shield students from experiencing the negative results of excessive stress. These factors, such as self-efficacy and optimism, have been shown to enhance individuals’ ability to cope with challenges (Bresó et al., 2011; Reed, 2016). Biggs et al. (2023) developed a psychological endurance model with grit and hardiness as the two core components of the psychological battery, which is confined partly by stressors from the environment. Grit, defined as the persistence of interest and effort towards a long-term goal (Duckworth et al., 2007), has been shown to have a protective nature against academic stress, with studies indicating that grittier students were less vulnerable to its effects (Mosanya, 2021). The unique features of grit, which involves sticking to long-term goals despite challenges, makes it stand out as a crucial psychological resource for overcoming academic stress and setbacks and preventing burnout (Teuber et al., 2021). Furthermore, additional findings suggested that both facets of grit protect students with high levels of burnout from experiencing depressive symptoms (X. Tang et al., 2021).

However, as a relatively new concept, studies with the purpose of improving grit levels among students are limited. Existing interventions that have been implemented within the school setting adopted various approaches. For instance, Alan et al. (2019) successfully increased students’ level of grit through a tailored, minimum 10-session weekly curriculum. This curriculum emphasized themes such as the malleability of the brain, the importance of effort and goal setting, and the constructive understanding of failures. Notably, the emphasis on the plasticity of one’s brain and ability, which is the core of the growth mindset, has been observed in other grit-enhancing studies as well (Mohammadi et al., 2023; Zappala-Piemme et al., 2023). On the other hand, some interventions for promoting a growth mindset also incorporated the concept of grit as part of the outlines, such as highlighting effort when encountering setbacks (Orosz et al., 2017). The intertwined focus between grit and a growth mindset can be traced back to evidence supporting the reciprocally predictive power between the two constructs (Park et al., 2020).

Based on the existing studies and suggestions proposed by Hwang and Nam (2021) regarding possible strategies for developing grit, the current study designed a 6-session grit-enhancing program. Previous research supports the effectiveness of such grit-building intervention schemes proposed by Hwang and Nam (2021). Shafiee Rad and Jafarpour (2023) successfully implemented a program with proposed topics among second language learners. Their findings indicated that the intervention not only enhanced grit but also fostered positive emotions.

The primary objective of this study was to test the effectiveness of grit-enhancing and mandala-coloring interventions in reducing academic stress and burnout among adolescents. This study hypothesized that (1) the grit-enhancing intervention would lead to a significant increase in grit along with a decrease in academic stress and burnout compared to other groups and (2) the mandala-coloring group would show a significant reduction in academic stress and burnout compared to other groups.

## 2. Materials and Methods

### 2.1. Study Design

A quasi-experimental design with pretest–posttest assessments was used.

### 2.2. Participants

The target population of this study consisted of 8th-grade students in a public middle school in a rural area in Henan Province. Cluster sampling was used to identify two 8th-grade classes, totaling 130 students. A total of 2 students failed to complete the post-intervention survey, which resulted in a final sample of 128 students.

### 2.3. Procedures

**Figure 1:**
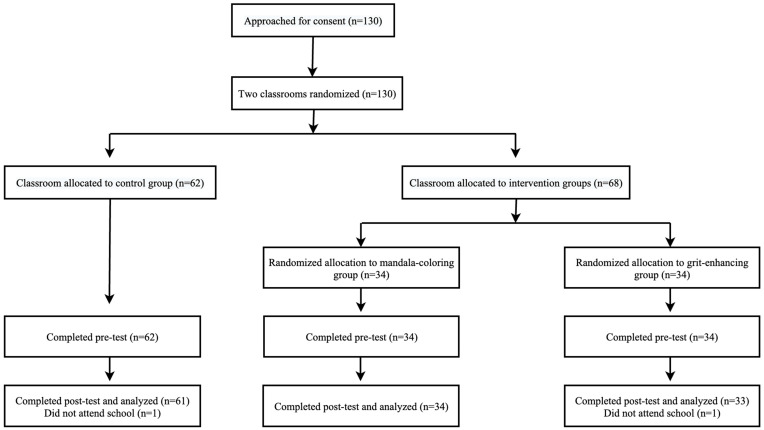
Participation flowchart.

In the two participating classes, one class was randomly assigned to the control group (*n* = 62), while the other class was assigned to the intervention group (*n* = 68). Within the intervention group, students were randomly divided into two sub-groups participating in different programs: the mandala coloring (*n* = 34) and the grit enhancing (*n* = 34) (see Figure 1). At the time of assignment, no baseline differences between groups were known. Any pre-existing differences were identified later during data analysis and are reported in the results. After the random allocation, participants first filled out the pre-intervention survey measuring their level of grit, academic stress, and burnout. Gender, age, parents’ income, and educational level were also collected as demographic information. Participants in the three groups were required to complete either a mandala-coloring program, a grit-enhancing program, or continue with their daily routine. Both of the two interventions lasted for 10 days with 6 sessions, and the distribution of the sessions was on day 1, day 3, day 5, day 7, day 9, and day 10. The instructor (first author) met with participants on the above six days to deliver the prepared intervention content. On day 10, participants were gathered together to finish the post-intervention survey and compensated with a stationery gift set. The intervention was approved by the research ethics committee of the first author’s university. Participation was entirely voluntary, and written consent was obtained from the participating students and their parents.

#### 2.3.1. Mandala-Coloring Program

**Figure 2:**
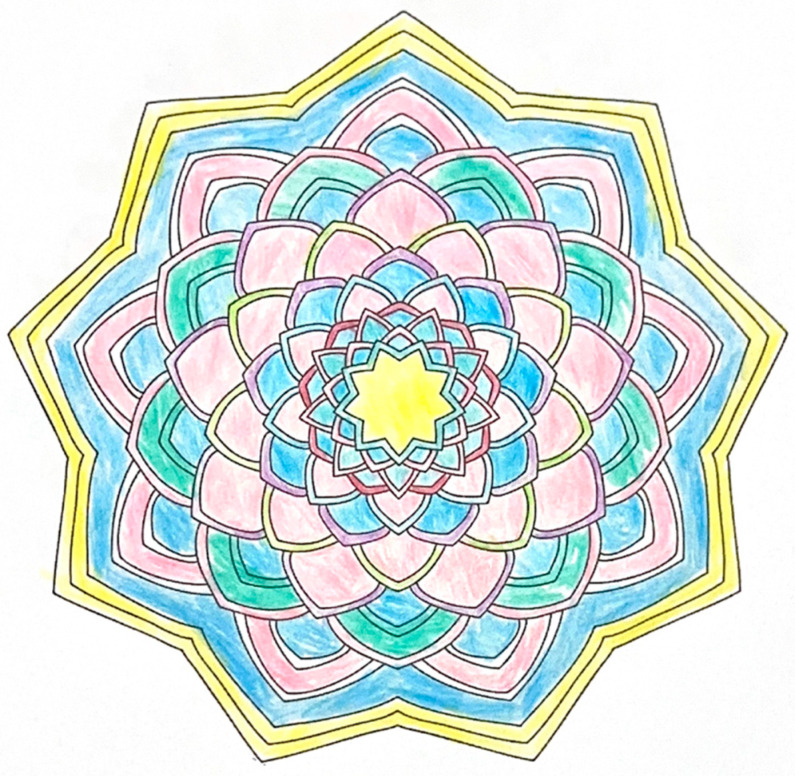
An example of a mandala coloring completed by participants.

Each participant was provided with a coloring book and a box of 24 coloring pencils. The coloring book included 11 mandala coloring patterns, from which participants were granted the freedom to select any 6 patterns to color over the course of 6 sessions. Participants had 30 min to finish their coloring independently in every session. The instructor introduced the concept of mandala drawing, accompanied by illustrative examples and step-by-step instructions on coloring techniques in the first session. Participants completed one piece of drawing in each scheduled subsequent session independently (Figure 2).

#### 2.3.2. Grit-Enhancing Program

This program was developed by utilizing various strategies to promote grit, as suggested by Hwang and Nam (2021). Each session had a duration of 30 min and was conducted concurrently with the mandala-coloring group, albeit in a different classroom. Table 1 summarizes the details of each session of the two intervention groups with different topics and activities.

### 2.4. Measures

#### 2.4.1. Academic Stress

Academic stress was measured by using one of the Chinese local scales (Y. Liu & Lu, 2012), which assesses stress related to homework demands, learning difficulties, and exam pressure. The scale has been used in the Chinese educational context, demonstrating good reliability and validity (e.g., Wang et al., 2020). Each item was rated on a 4-point Likert-type scale (1 = strongly disagree; 4 = strongly agree), with higher scores indicating greater academic stress. An example item is “To finish my homework makes me feel pressure”. This scale demonstrated a good internal consistency, Cronbach’s α = 0.84.

#### 2.4.2. Academic Burnout

The Chinese version of the Maslach Burnout Inventory-Student Survey (MBI-SS; Hu & Schaufeli, 2009) was applied to measure the level of academic burnout. The scale has been tested as valid across different age groups in the context of China (C. Li et al., 2021). The MBI-SS includes three subscales corresponding with three dimensions of academic burnout: exhaustion, cynicism, and inefficacy. Example items are, respectively: “Studying or attending a class is really a strain for me”, “I doubt the significance of my studies”, and “I feel stimulated when I achieve my study goals”. Based on a 7-point Likert-type scale (0 = never; 6 = always), the higher scores indicate a severer degree of burnout. The scale demonstrated a good internal consistency in this study, Cronbach’s α = 0.90.

#### 2.4.3. Grit

The Chinese version of the Short Grit Scale (J. Li et al., 2018) was used to assess the participants’ grit level. Perseverance of effort and consistency of interest are the two dimensions included in the Short Grit Scale, and the example questions are: ” I finish whatever I begin”, and “New ideas and projects sometimes distract me from previous ones”. Respondents rated each item on a 5-point Likert-type scale (0 = not at all like me; 6 = very much like me), with higher scores suggesting a higher level of overall grit. The scale demonstrated a decent internal consistency in this study, Cronbach’s α = 0.65.

### 2.5. Statistical Analyses

All data analyses in this study were performed using IBM SPSS, version 26. Descriptive statistics were used to summarize demographic characteristics, with independent samples *t*-tests and χ^2^ tests performed to detect any baseline differences between groups. One-way ANOVAs were conducted to compare the baseline data between groups.

To evaluate the effectiveness of the interventions, ANCOVA was conducted with pre-test scores as covariates to control for baseline differences. Effect sizes (partial η^2^p) were reported to assess the practical significance of observed differences.

For within-group changes, a repeated measures ANOVA was performed to analyze the effect of time on academic stress, burnout, and grit levels. A *p*-value of <0.05 was considered statistically significant.

## 3. Results

### 3.1. Preliminary Analyses

As Table 2 shows, there were no significant differences in demographic information among the three groups. The mean age of the participants was 13.48 years old, and 54 (42.2%) were girls. However, for pre-test scores, the variable of burnout exhibited a statistically significant difference, *F* = 3.317 and *p* < 0.05, with considerably higher scores observed in both the grit-enhancing group and control group (see Table 3). Thus, ANCOVA was selected for subsequent analyses over alternative statistical methods, in accordance with Kenny’s (1979) recommendation, as it is deemed appropriate when baseline data may present potential challenges related to group differences.

### 3.2. Effects of Interventions

A series of ANCOVAs were conducted to explore any statistical difference between the mandala-coloring group, grit-enhancing group, and control group on the measures at post-test, controlling the pre-test scores as covariates. The results suggest that there was a significant difference between groups on the stress scale, *F* = 5.741, *p* = 0.004, and η^2^p = 0.085. Post hoc tests revealed the significant differences in stress levels that exist between the mandala-coloring group and control groups (*p* < 0.05). For burnout, there was no significant difference between groups, *F* = 1.15, *p* = 0.32, and η^2^p = 0.018. For grit, there was no significant difference between groups, *F* = 1.794, *p* = 0.171, and η^2^p = 0.028 (see Table 4).

**Figure 3:**
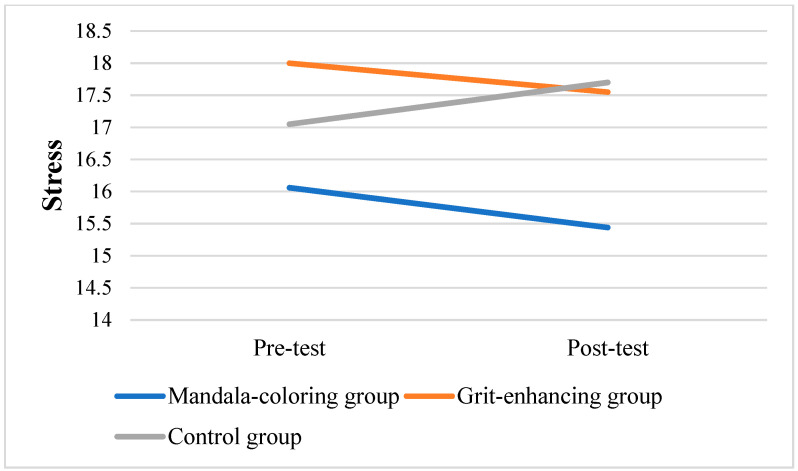
Changes in stress scores among three groups.

**Figure 4:**
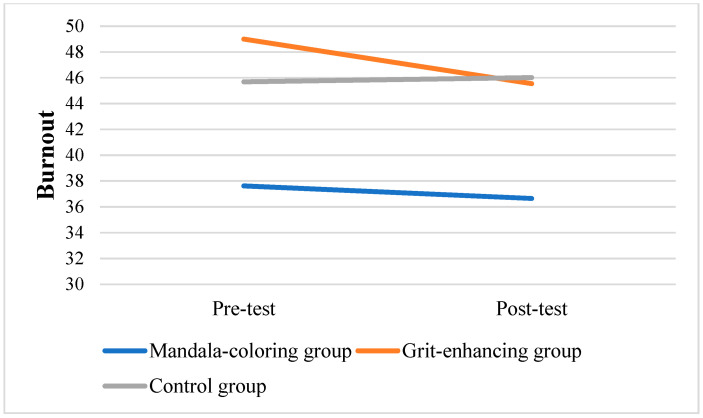
Changes in burnout scores among three groups.

**Figure 5:**
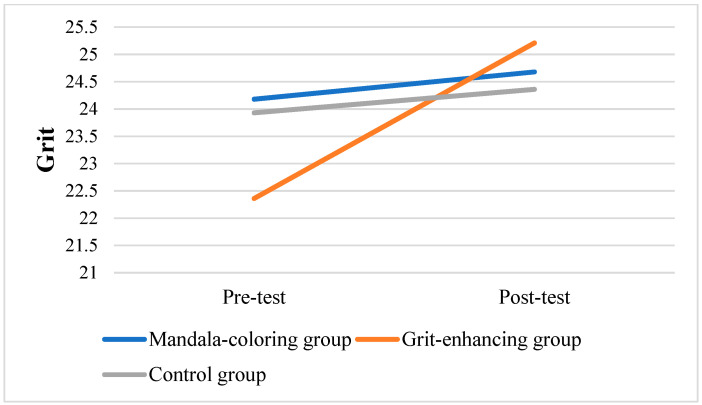
Changes in grit scores among three groups.

To further assess within-group changes, repeated measures of ANOVAs were performed to analyze variations from pre-test to post-test in the three groups. Results showed that only in the grit-enhancing group, the grit level significantly increased from pre-test to post-test scores (*F* = 16.925, *p* < 0.001). Figure 3, Figure 4 and Figure 5 displayed the changes in target variables from the pre-test to post-test scores.

## 4. Discussion

The current study aimed to test two interventions, namely mindfulness mandala coloring and building grit as a shielding factor, for reducing academic stress and burnout among middle school students. This study found that the participants in the mandala-coloring group exhibited a significant decrease in the level of stress between groups. However, the grit-enhancing group did not demonstrate a significant increase in grit levels nor a decrease in stress levels between groups. The findings on the influence of coloring a symmetric shape, such as a mandala, are consistent with previous research that the therapeutic effect of mandalas could relieve academic stress for students (Apriyana et al., 2020), even over a short duration such as one week. Carsley and Heath (2018) found that a single session of mandala coloring led to immediate reductions in the anxiety state among adolescents, with participants reporting increased feelings of full concentration. Similarly, Babouchkina and Robbins (2015) showed that structured coloring activities, particularly mandala designs, facilitated greater emotional regulation compared to free coloring or unstructured artistic tasks. However, despite its impact on stress, no significant reduction in burnout levels was observed between groups. While some studies have reported that mindfulness-based practices significantly reduce both stress and burnout (e.g., Green & Kinchen, 2021; Ireland et al., 2017), similar findings to the present study have also been documented. For instance, Suyi et al. (2017) conducted a six-week mindfulness intervention for mental health professionals in Singapore and found a significant decrease in the levels of stress, yet burnout levels remained unchanged. According to the Job Demands–Resources (JD-R) Model, burnout is the outcome of a complex developmental process influenced by the interplay of job or study demands and resources (Bakker & Demerouti, 2007). Therefore, changes in burnout may require an extended period to manifest. Additionally, the research suggests that stress could predict burnout through various internal and external factors, such as self-efficacy, cognitive appraisal, as well as sleep quality (Fariborz et al., 2019; Gomes et al., 2013; Song et al., 2020), suggesting the importance of taking these factors into account when assessing changes in the burnout level.

Although the grit-enhancing group did not yield any statistically significant between-group changes, the grit level within this group did increase significantly. Figure 5 illustrates a noticeable increase in grit levels, accompanied by a slight decrease in both stress and burnout levels. These findings strengthened the prior research, such as the study by Sigmundsson and Hauge (2023), which observed that even a single-session online intervention led to notable increases in grit levels.

Several potential explanations exist for the lack of between-group significant changes. Firstly, the program’s duration and participant numbers might be insufficient for achieving statistical significance. Programs focusing on mindset development with similar numbers of sessions are typically conducted weekly for at least 5 weeks to observe significantly positive outcomes (Orosz et al., 2017). So, outstretching the duration of the program may yield some more distinct results. Another reason for the absence of significant outcomes is the occurrence of “unhappy randomization” in the first place. Participants in this group seemingly displayed higher stress and burnout levels with a lower level of grit (refer to Table 4). This initial imbalance may have influenced the following data-processing part which lowered the significant variance of the final results. Additionally, factors related to session delivery may have influenced the results. Previous research suggests that school-based interventions are most effective when delivered by teachers rather than external facilitators, as students tend to feel a stronger sense of connection and engagement (Carsley et al., 2018). Nevertheless, the significant increase in the level of grit within the group suggested that the effectiveness of the designed program deserves further investigation.

### 4.1. Limitations

This study aims to test the effectiveness of two interventions on mitigating stress and burnout, with one focusing on directly reducing stress and another one through the channel of building grit. Several limitations need to be noted. First, participants in this study were exclusively from the rural area of Henan province, precluding the generalization of conclusions to diverse samples. Second, as aforementioned, the design’s quasi-experimental randomization performed on a class level initially may have negatively affected the outcomes of this study. Third, the utilization of cluster sampling, employing an entire class as the control group, resulted in an uneven distribution among the groups. Fourth, the study’s limited sample size and short duration may have contributed to some of the insignificant results. Future studies can address some of the limitations by incorporating a more diverse participant pool and employing refined sampling techniques.

### 4.2. Conclusions

To sum up, this study investigated the efficacy of school-based interventions utilizing mandala-coloring and grit-enhancing techniques in mitigating academic stress and burnout among Chinese adolescents. The findings shed light on the potential benefits of integrating mindfulness coloring and resilience-building strategies into school curriculums to support students’ mental well-being. Specifically, mandala-coloring interventions show promise as effective tools for alleviating the negative effects of academic stress, while the impact of grit-enhancing interventions requires further investigation. Mandala coloring serves as a convenient and impactful tool for reducing academic stress, particularly due to its time-efficient nature, making it well suited for the demanding academic schedules of middle and high school students. Additionally, it requires minimal teacher training, as evidenced by prior studies, such as Carsley and Heath (2019), which highlight the accessibility of such mindful art activities. Therefore, it is recommended that schools incorporate mindfulness-based mandala coloring as an after-class art activity to support students’ well-being. However, the grit-enhancing intervention may require more structured guidance from experienced professionals.

These findings underscore the importance of addressing the psychological well-being of students in educational settings and highlight the value of incorporating holistic approaches to promote stress-coping skills. Moving forward, further research and implementation efforts are warranted to fully understand the long-term impacts of these interventions and to develop comprehensive support systems for adolescent mental health within school environments.

## Figures and Tables

**Table 1:** Summary of mandala-coloring and grit-enhancing programs.

Session	Mandala-Coloring Group	Grit-Enhancing Group
1	Choose a mandala to color by yourself	Topic: Mindset Brief introduction: a short quiz to test one’s mindset; introduce the concept of mindsets through a video about growth mindset and fixed mindset (5 min); compare two types of mindsets in terms of characteristics; use a brainstorming game to further reinforce different concepts of thinking patterns: How would individuals with different mindsets think and behave in different scenarios? Summarize.
2	Choose a mandala to color by yourself	Topic: Mindset Play the game “Words spelling contest” to introduce the theme of brain’s ability to develop; read the article “You Can Grow Your Intelligence” and discuss in groups: What have you learned from this article? What aspect of growth mindset do you find most interesting after two sessions? Summarize.
3	Choose a mandala to color by yourself	Topic: Power of failure Introduce the concept of failure through a word guessing game; review the words that you failed to guess correctly, and discuss how to define failure; reflect and write about a past experience of failure, share the experience in pairs, and then discuss: How did you overcome this failure/setback? Did this failure have any positive impact on you? Learn about Michael Jordan’s example of “learning from failure.” Summarize.
4	Choose a mandala to color by yourself	Topic: SMART goal setting Recall this year’s New Year resolutions and have you accomplished yet; share a short story highlighting goal setting; understand what SMART (specific, measurable, actionable, relevant, time-bound) goals are through a video; individually reflect and set a new goal (in any area, such as learning a new skill, maintaining a fitness routine, obtaining better grades in the next exam, etc.), and share. Summarize
5	Choose a mandala to color by yourself	Topic: The role of effort and self-regulation Introduce the importance of effort through a card stacking game; introduce the marshmallow experiment and watch a video; reflect on the purpose of this experiment; discuss the differences between children who were able to resist the temptation and those who were not. Review the SMART goal setting in the previous class and discuss how to use effort and self-control to achieve your goals. Summarize.
6	Choose a mandala to color by yourself	Topic: Emotion regulation Introduce how we perceive and process emotions, and how mandala drawing can help us manage emotions and stress; distribute one mandala drawing, finish it within 20 min. Summarize.

**Table 2:** Participants’ demographic information.

Variables	Mandala-Coloring Group (*N* = 34)		Grit-Enhancing Group (*N* = 33)		Control Group (*N* = 61)		Test	*p*
	N	%	N	%	N	%		
Gender							*χ*^2^(2) = 0.20	0.99
Female	14	41.2	14	42.4	26	42.6		
Male	20	58.8	19	57.6	35	57.4		
Parents’ income (RMB)							*χ*^2^(8) = 8.73	0.37
<3500	7	20.6	7	21.2	13	21.3		
3500~7000	20	58.8	16	48.5	28	46.0		
7000~10,000	6	17.7	7	21.2	17	27.8		
10,000~15,000	0	0.0	3	9.1	3	4.9		
≥15,000	1	2.9	0	0.0	0	0.0		
Parents’ education level							*χ*^2^(6) = 6.73	0.35
Middle school graduated	24	70.6	27	81.8	44	72.1		
High school graduated	6	17.6	5	15.2	14	30.0		
Bachelor’s degree	2	5.9	1	3.0	3	4.9		
Master’s degree	2	5.9	0	0.0	0	0.0		
Age (Mean ± *SD*)	13.65 ± 0.69		13.42 ± 0.56		13.41 ± 0.53		*F*(2) = 1.98	0.14

**Table 3:** Baseline scores in three groups (Mean ± SD).

Variable	Groups			*F*	*p*
	Mandala-Coloring Group	Grit-Enhancing Group	Control Group		
Stress	16.06 ± 2.73	18.00 ± 3.57	17.05 ± 3.37	2.858	0.061
Burnout	37.62 ± 13.16	49.00 ± 19.89	45.69 ± 20.87	3.317	0.039
Grit	24.18 ± 4.13	22.36 ± 4.54	23.93 ± 5.16	1.519	0.223

**Table 4:** Changes in scores (Mean ± SD).

Variable	Mandala-Coloring Group		Grit-Enhancing Group		Control Group		*F*	*p*	η^2^p
	Pre	Post	Pre	Post	Pre	Post			
Stress	16.06 ± 2.73	15.44 ± 2.41	18.00 ± 3.57	17.55 ± 3.54	17.05 ± 3.37	17.70 ± 4.15	5.741	0.004	0.085
Burnout	37.62 ± 13.16	36.65 ± 12.47	49.00 ± 19.89	45.55 ± 20.37	45.69 ± 20.87	46.02 ± 22.25	1.150	0.320	0.018
Grit	24.18 ± 4.13	24.68 ± 4.39	22.36 ± 4.54	25.21 ± 5.22	23.93 ± 5.16	24.36 ± 6.85	1.794	0.171	0.028

## Data Availability

The data that support the findings of this study are available from the corresponding author upon reasonable request.
